# Intrapericardial fibrinolysis in purulent pericarditis—case report

**DOI:** 10.1186/s12245-015-0087-y

**Published:** 2015-10-07

**Authors:** Małgorzata Dybowska, Barbara Kazanecka, Paweł Kuca, Janusz Burakowski, Cezary Czajka, Franciszek Grzegorczyk, Renata Gralec, Witold Tomkowski

**Affiliations:** Cardio-Pulmonary Intensive Care Department, National Institute of Tuberculosis and Lung Diseases, ul. Płocka 26, 01-138 Warsaw, Poland

**Keywords:** Purulent pericarditis, Constrictive pericarditis, Subxiphoid pericardiotomy, Fibrinolysis

## Abstract

Purulent pericarditis (PP) continues to result in a very serious prognosis and high mortality. The most serious complication of pericarditis is constriction. Intrapericardial administration of fibrinolytic agents, although controversial, can prevent the development of constrictions. We present the case of a 63-year-old man with purulent inflammation of the right knee who was admitted to the intensive care unit (ICU) via emergency room orthopedic evaluation because of purulent pericarditis. Subxiphoid pericardiotomy was urgently performed, with 1200 ml of thick purulent fluid evacuated. As prevention for pericardial constriction, it was decided to administer fibrinolysis to the patient’s pericardial cavity. Administration of streptokinase was complicated by the occurrence of a severe retrosternal pain and intrapericardial bleeding. Due to insufficiency of antibiotic therapy, 17 days after complicated fibrinolytic therapy with streptokinase, it was decided to administer 20 mg of r-tPA directly into the pericardium. In the following days, there remained a high drainage of purulent secretions. Fever up to 38 °C was still observed despite the use of antibiotics. Nine days after first administration of r-tPA, it was decided to apply the next dose. Daily drainage decreased from 50 to 20 ml in successive days. No fluid accumulation and symptoms and signs of constrictions were observed in clinical examinations as well as in echocardiography performed during 7 years follow-up after discharge.

## Background

Purulent pericarditis (PP) continues to result in a very serious prognosis and high mortality. Untreated, it always leads to death, and even after proper multidirectional treatment, around 40 % of patients still die [1]. The following factors are predispositions to the development of PP: the presence of fluid in the pericardium, immunosuppression, chronic illness, surgeries, and injuries of the chest. The disease usually occurs suddenly, with a high fever and serious general condition of the patient. Most commonly, PP is accompanied by the presence of inflammation, which is the source of bacteria settling in the pericardial cavity, which can get into the pericardium via hematogenous spread, initially causing bacteremia or contiguous. The most common microorganisms gained from fluid cultures are *Staphylococci*, *Pneumococci*, *Streptococci*, *Proteus*, *Neisseria*, Gram-negative bacteria, and *Legionella*. PP is characterized by a high white blood cell count in the fluid exceeding 10,000/ml, and smear fluid granulocytes and macrophages predominate. The liquid is characterized by a high protein concentration. PP can lead to cardiac tamponade and/or septic shock, due to which any suspected purulent pericarditis always requires urgent surgery with placement of a drain into the pericardium, and rinsing with saline solution and an appropriate, broad-spectrum antibiotic used intravenously. This should be implemented even before the results of bacteriological tests, preferably after downloading material (pericardial fluid and blood) for temporary microscopic examination and cultures [2]. In the initial antimicrobial therapy, an antistaphylococcal drug should be used in combination with an aminoglycoside, and after obtaining the culture results, the treatment should be modified accordingly. Diagnosis of PP should result in urgent use of targeted antibiotics as well as early surgical intervention (subxiphoid pericardiothomy), which improve prognosis. The most serious complication of pericarditis is constriction. Constrictive pericarditis is characterized by the loss of elasticity of the pericardium by its atresia, progressive fibrosis, and eventually calcification. Reducing the flexibility of the pericardium impairs blood flow to the heart in the diastole, causing an increase in the filling pressure and gradually increasing heart failure. Clinching of the pericardium may develop even in a more distant period after completion of a period of acute pericarditis, hence the need to monitor patients after leaving the hospital to perform early pericardiectomy surgery if necessary. Surgical treatment of patients with pericardial thickening and tightening characteristics exposes the patient to the possibility of complications and involves quite a high mortality rate of about 8 %. Intrapericardial administration of fibrinolytic agents, although controversial, probably prevents the development of constrictions [3–6].

## Case presentation

**Fig. 1:**
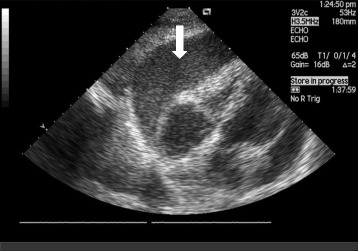
Echocardiography: hyperechogenic fluid around the whole heart (*arrow*)

A 63-year-old man with purulent inflammation of the right knee was admitted to the intensive care unit (ICU) in January 2008 due to suspected purulent pericarditis. The patient was not treated chronically. In an interview, he revealed that for many years, he suffered from pain of the fingers, hands, feet, and spine. He was never rheumatologically diagnosed. He temporarily received non-steroidal anti-inflammatory drugs. A few weeks before admission to the ICU, a pain in the right knee appeared in the patient, followed by fever. In the course of orthopedic diagnosis, a knee puncture was performed, confirming its purulent inflammation. In the synovial fluid culture, a methicillin-susceptible strain, *Staphylococcus aureus*, was grown and antibiotic therapy was implemented in accordance with the culture (cefazolin, ciprofloxacin, metronidazole). During orthopedic diagnosis, the patient experienced arrhythmias in the form of paroxysmal atrial flutter. The echocardiography showed exudative pericarditis and the presence of hyperechogenic fluid around the whole heart, the right ventricle to 3.6 cm, and the apex to 3.7 cm (Fig. 1).

Purulent pericarditis was suspected in the patient with suppurative arthritis, and the patient was transferred to the ICU. On admission to the ICU, the patient was in guarded condition. Abnormalities observed were edema of the low extremities, with an irregular heart rate of 110/min., and BO 130/70 mmHg.

**Fig. 2:**
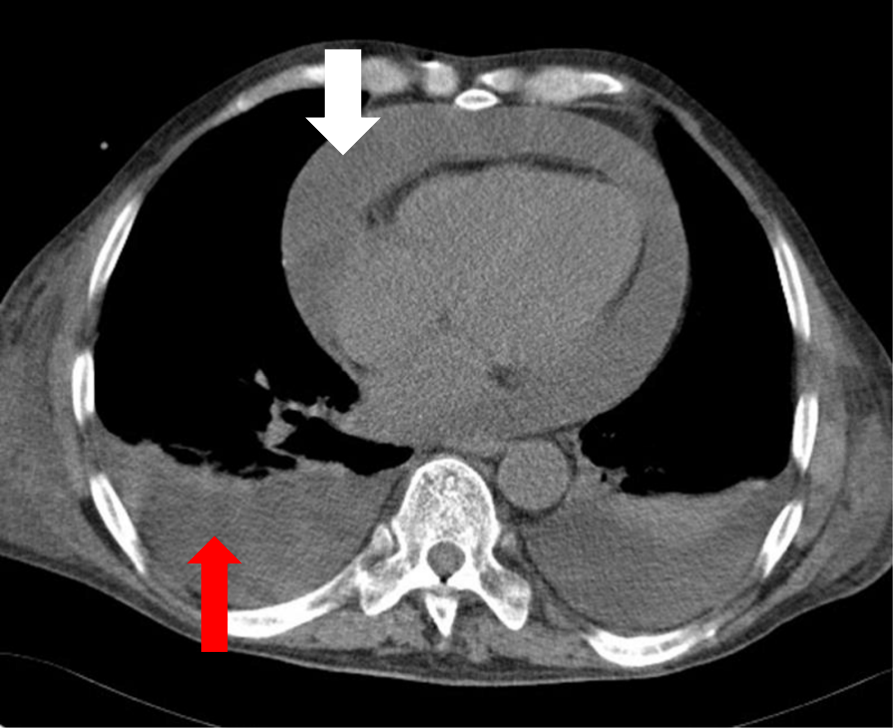
CT of the chest: *white arrow* pericardial effusion, *red arrow* pleural effusion

CT of the chest was performed, which confirmed a very large amount of fluid in the pericardium (Fig. 2).

A pericardial puncture procedure was urgently performed, with 1200 ml of thick purulent (confirmed by macroscopic examination) fluid evacuated. A large drain was placed in the pericardial cavity.

The results of the general test of the fluid are in the Table 1.

**Table 1:** The results of the general test of the fluid

Color	Very turbid
pH	5
Weight	1025
Protein	0.8 g %
Glucose	72 mg %
Cholesterol	164 mg %
LDH	5492 U/l

The results of the fluid-microscope smear are in the Table 2.

**Table 2:** The results of the fluid-microscope smear

Leukocytes	65 × 10^9^
Neutrophils	66 %
Lymphocytes	15 %

Multiple cultures of the pericardial fluid, blood, and a pericardial slice culture, including testing for tuberculosis, were negative. The next day after surgery on the pericardial cavity, the patient drained 150 ml of sanguine-purulent fluid. In a routine echocardiography, heart wall thickening of the pericardium to 5 mm was established. As a prevention of pericarditis construction, it was decided to provide fibrinolysis to the patient’s pericardial cavity. Three doses of streptokinase, 500,000 IU each, were scheduled every 12 h. Streptokinase was dissolved in 50 ml of 0.9 % NaCl and was administered by a catheter to the pericardial cavity. After administration of the drug, the drain was clamped for 12 h. The first administration of streptokinase was complicated by the occurrence of a severe retrosternal pain. The patient drained 250 ml of sanguinary purulent fluid. Further doses of the drug were discontinued. In view of the persistent purulent pericardial drainage up to 150 ml for 2 weeks, despite the use of broad-spectrum antibiotics (piperacillin/tazobactam, ciprofloxacin, linezolid), echocardiography was performed. This showed features of growing constriction of the pericardium with a progressive thickening of the pericardium blades to 8–10 mm, with visible material allowance echoes both on the pericardium and the visceral wall. Pericardial plaques were strong, saturated, and stiffer. The patient had a fever up to 38 °C. Due to insufficiency of antibiotic therapy, 17 days after complicated fibrinolytic therapy with Streptokinase, it was decided to administer r-t PA directly into the pericardium. Actilyse (20 mg) was dissolved in 100 ml of solvent and administered by a catheter into the pericardium. Pericardial drain was clamped for 24 h. The next day, the patient drained 300 ml of pericardial fluid. In the following days, there remained a high drainage of purulent secretions: 250, 100, 100, 50, and 50 ml. Fever was still observed despite the use of antibiotics (piperacillin/tazobactam, linezolidum, meropenem). Nine days after first administration of Actilyse, it was decided to give the next dose. The treatment consisted of 20 mg Actilyse in 50 ml of solvent. The drain was clamped for 24 h. Daily drainage decreased from 50 to 20 ml in successive days. Also, the fever subsided. At 6 days after the second r-tPA dose, the pericardial drain was removed. The total volume of purulent fluid drained was very large and exceeded 4000 ml. There were no complications of the treatment, with no significant deviations in coagulation parameters (thrombin time and INR) and peripheral blood counts. No fluid accumulation and symptoms and signs of constrictions were observed in clinical examinations as well as in echocardiography performed during 7 years follow-up after discharge.

## Discussion and conclusions

In our knowledge, this is the first report of a patient with PP treated with two different fibrinolytic agents administered directly into pericardial space. In our opinion, indications for administration of fibrinolytic agents into the pericardial space are: loculation of the pericardial space and thickening of pericardium, which are associated with hindered penetration of the antibiotic into the pericardium. The good clinical effect of this form of treatment is probably due to dissolution of pathological fibrin deposits connecting the epicardium to the pericardium, which also allows a better penetration of antibiotics. Clinical observations based on a small group of patients suggest that the administration of fibrinolytic drugs intra pericardium is an effective way to prevent the development of pericardial constriction and thus protects the patient from having to perform pericardiotomy. Up to now, no randomized studies, confirming the effectiveness or efficiency of this treatment, have been performed. So far, the most effective and safe dose of fibrinolytic agent administered directly into the pericardial space is unknown as well as the regiment of treatment (number of doses, intervals between doses, the most effective agent).

## Consent

Written informed consent for the case report and any accompanying images were obtained. A copy of the written consent is available for review by the Editor-in-Chief of this journal.

## Abbreviations

ICU: intensive care unit
PP: purulent pericarditis
